# Supplementary material for: An Odor Labeling Convolutional Encoder–Decoder for Odor Sensing in Machine Olfaction
Source: Sensors (Basel). 2021 Jan 8;21(2):388. doi: 10.3390/s21020388 (PMC7826699; doi:10.3390/s21020388)
Supplement: Supplementary file 1 [file sensors-21-00388-s001.pdf]

Supplementary document: Accuracy and Loss Rate in training

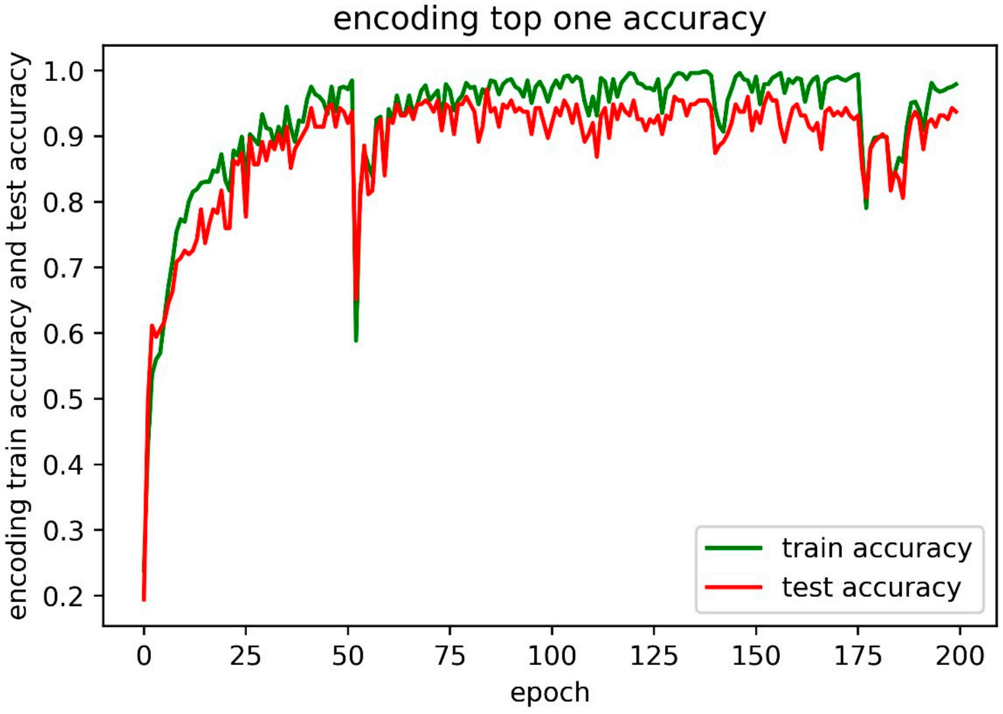

Figure S1. The accuracy of the top1 model in training.

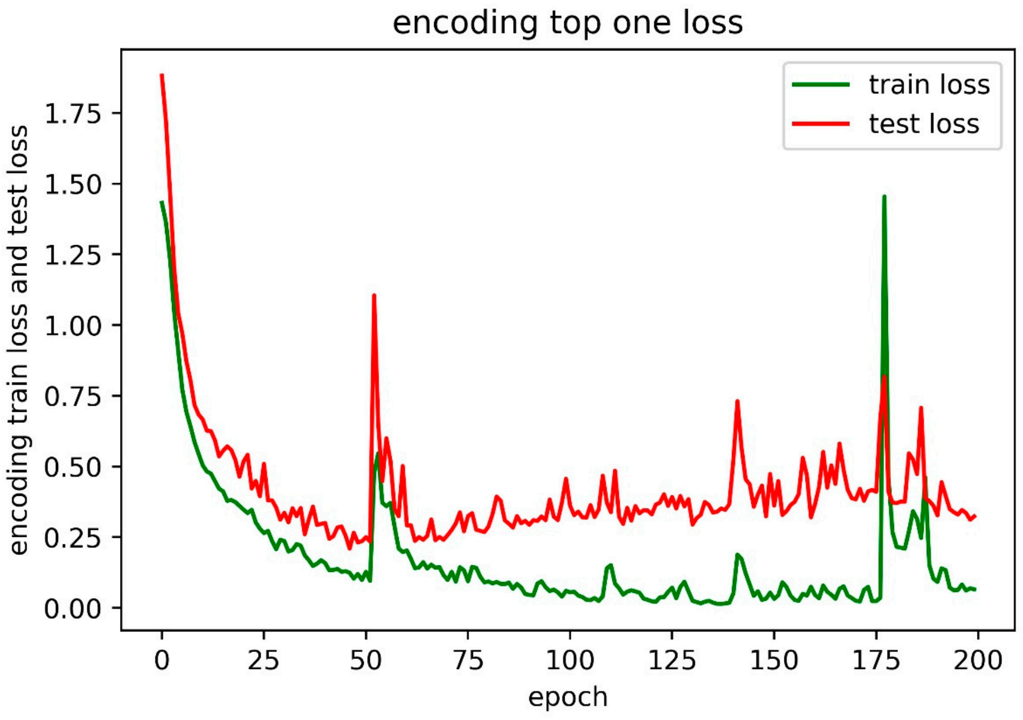

Figure S2. The loss rate of top1 model in training.

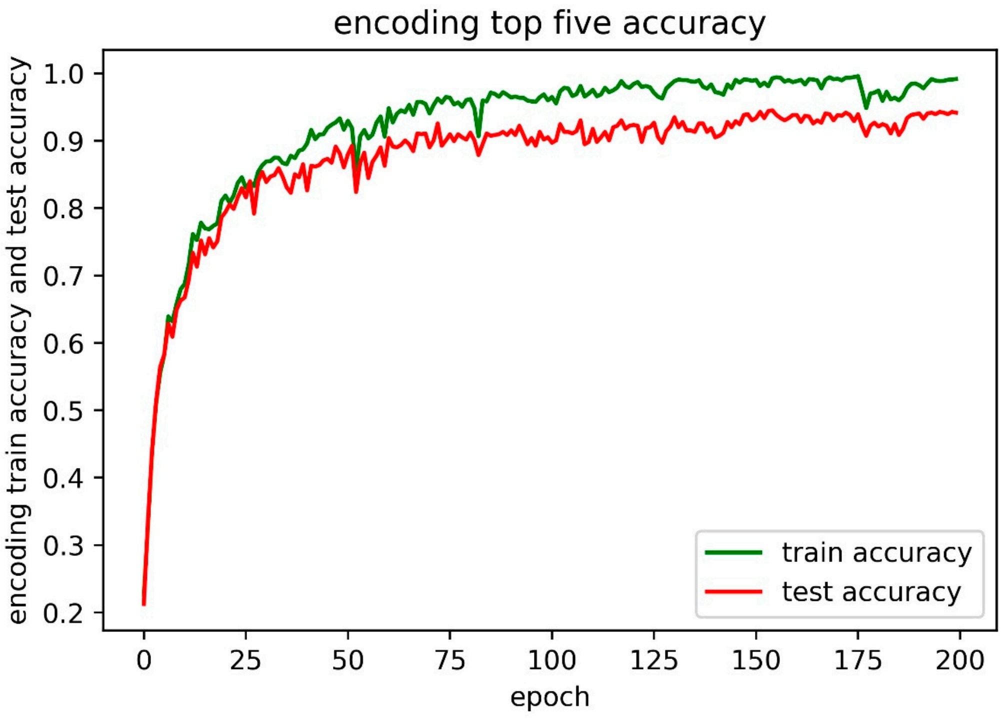

Figure S3. The average accuracy of top five models in training.

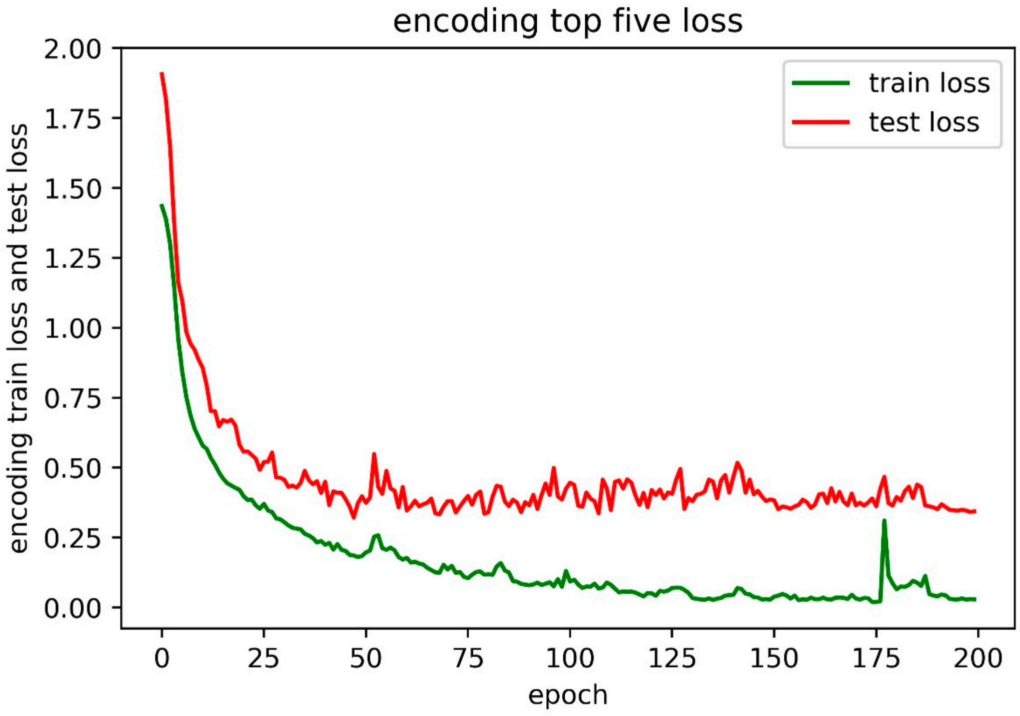

Figure S4. The average loss rate of top five models in training.
